# Supplementary material for: Compositional dependence of the fragility in metallic glass forming liquids
Source: Nat Commun. 2022 Jun 28;13:3708. doi: 10.1038/s41467-022-31314-3 (PMC9240049; doi:10.1038/s41467-022-31314-3)
Supplement: Supplementary file 1 — Supplementary Information [file 41467_2022_31314_MOESM1_ESM.docx]

Supplementary Information

**Compositional dependence of the fragility in metallic glass forming liquids**

Sebastian A. Kube^1^, Sungwoo Sohn^1^, Rodrigo Ojeda-Mota^1^, Theo Evers^1^, William Polsky^1^,

Naijia Liu^1^, Kevin Ryan^2^, Sean Rinehart^3^, Yong Sun^3^, Jan Schroers^1,*^

^1^ Department of Mechanical Engineering and Materials Science, Yale University, New Haven CT, United States.

^2^ School of Engineering and Applied Science, Yale University, New Haven CT, United States.

^3^ Department of Applied Physics, Yale University, New Haven CT, United States.

*Corresponding author. E-mail address: jan.schroers@yale.edu

**Supplementary Methods**

**Sample preparation:** Double side polished (100) silicon wafers (Pure Wafer Inc.) of 550 µm thickness were used. The front was coated with a 10 nm sacrificial layer of amorphous dry thermal SiO_2_ (FirstNano CVD), which in a later step served as etch-stop during DRIE and is subsequently removed using an HF vapor etch. The wafers were primed using HMDS (hexamethyldisilazane). To prepare the etch mask, the back side was spin-coated with 0.5 µm lift-off resist (LOR A5, Kayaku Advanced Materials, Inc.), followed by 0.5 µm positive resist (Shipley S1805, Kayaku Advanced Materials, Inc.). After 10 seconds of UV photoexposure using an EVG EV620 contact aligner, the resist was developed in MF319 developer (Kayaku Advanced Materials, Inc.). A layer of 150 nm Al_2_O_3_ was deposited using e-beam evaporation (Denton Vacuum, LLC). Following lift-off in N-Methyl-2-pyrrolidone (NMP, Thermofisher Scientific) at 80°C, the hard etch mask displayed in Figure 2.a.1 was obtained, and spin-coated with S1805 for protection during further processing. The mask features 5 mm square chips aligned with the (100)-wafer’s cleavage planes and separated by 300 µm wide grid lines. A 2 mm circular area of bare silicon is located at the center of each chip, for later etching down to the suspended metallic glass film.

In the next step, a resist-based lift-off mask was applied to the substrate’s front side prior to sputtering. This step is omitted in Figure 2 for brevity. It allows to add a small ID number feature (Supplementary Figure 1) to the frontside upper-left corner of each sample chip, at which the silicon is exposed rather than coated with the metallic glass film.


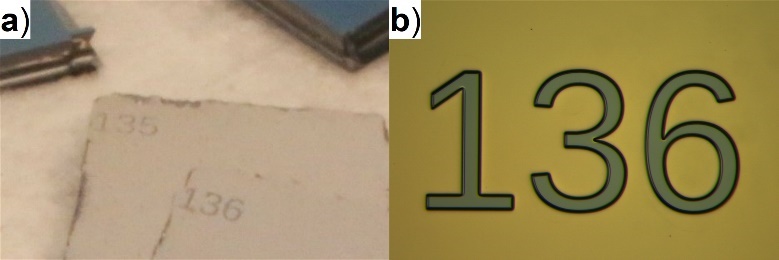


**Supplementary Figure 1:** ID number feature on front of chip for chip identification and film thickness measurement. **(a)** Photograph, **(b)** optical micrograph.

This feature serves a double purpose: Firstly, the ID number individually labels the chips from 001 to 241. Following the final cleaving step, it unambiguously identifies each individual chip, allowing for easy matching to the characterization results from EDX, XRD, and profilometry. Secondly, each digit provides step-features between the bare silicon surface and the top of the metallic glass film. These are used to locally measure the sputtered film thickness on each chip using profilometry. To apply this mask, the front side was first spin-coated with 4 µm lift-off resist (LOR A30, Kayaku Advanced Materials, Inc.), followed by 1.5 µm positive resist (Shipley S1813, Kayaku Advanced Materials, Inc.). After 3 seconds of photoexposure, the resist was developed in MF319 developer, leaving behind the unexposed resist patterned as small ID numbers with sufficient undercut for lift-off after sputtering.

In the next step, confocal DC magnetron co-sputtering (AJA International ATC2200) was used to deposit the compositionally graded metallic glass film. The sputtering sources are tetrahedrally arranged towards the substrate (cf. sputtering schematic in Figure 2). The sources were tilted to an angle of 37.3°, and the substrate stage was raised to the largest possible working distance of 22 cm. While this reduces the compositional spread obtained across the graded film (factor 2 compared to standard configuration), it simultaneously increases the density of compositional points covered by the ensemble of chips, thus increasing the compositional resolution of the overall method. The sputtering powers and accordingly the deposition rates were adjusted to yield the desired center compositions (Mg_65_Cu_25_Y_10_), deposition time was chosen to yield the desired center thickness (~1.35µm). Before deposition, the processing chamber was evacuated to a base pressure of 5.0E−7 Torr or less. The film was then deposited under flowing ultra-high purity argon at 5.8E−3 Torr. Target purities were 99.95% or better (Kurt Lesker Company). Following this deposition, lift-off was performed in NMP at 80°C, leaving behind bare silicon shaped as the ID number digits.

**Sample characterization:** In the next step, the compositionally graded film was characterized in high-throughput using customized automation routines controlling the respective xy-stages, providing individual measurements for each chip’s location on the wafer. The composition was measured using automated EDX (Helios G4 FIB-SEM with UltraDry EDX detector, 25 kV accelerating voltage, 40 secs live-time), and calibrated based on bulk samples of known compositions. X-ray diffractograms were acquired to identify amorphous or partially crystalline film (Rigaku Smartlab X-ray diffractometer, Bragg-Brentano focusing, Cu-Kα radiation, 5 mm beam mask, ~5 min per chip). Here, the substrate was tilted out of the detection plane by 10°, in order to eliminate the background signal from the single-crystalline substrate. The film thickness was measured by profiling the step heights at each of the ID-number features (Bruker Dektak XT stylus profilometer).

**Etching and cleaving:** In the final step, the substrate was subjected to deep reactive ion etching (DRIE) using the Bosch process (Oxford PlasmaPro100 DRIE) on the Al_2_O_3_-masked side. The vertical etch was performed throughout the silicon, down to the metallic glass film. The chips were then easily cleaved from the wafer along the etched gridlines. Finally, the chips were individually exposed to a 3-second HF vapor etch (49% Hydrofluoric Acid, KMG Electronic Chemicals, Inc.) in order to remove the sacrificial SiO_2_ layer. The HF did not visibly attack the Mg-Cu-Y film, which is attributed to the protective native oxide layer formed on these alloys (see following section).

**Effects of oxidation, substrate, and heating rate:** The effects of oxidation, substrate, and heating rate on the fragility values measured here are expected to be negligible. First and foremost, a fragility value of *m* = 45 was reported for Mg_65_Cu_25_Y_10_ in the literature ^1, 2^. This value was measured using bulk alloy samples in three-point beam bending under Argon inert gas conditions following structural equilibration under isothermal conditions at various temperatures. Within our own data, the closest matching composition is Mg_65.6_Cu_24.8_Y_9.6_. At less than 1 at.% compositional difference from the above literature composition, our measurement yields a fragility value of *m* = 44. This close agreement validates our method.

More specifically, Mg-Cu-Y glasses are widely known for their exceptional oxidation and corrosion resistance. This is attributed to a nanometer-thin, continuous, passivating magnesium oxide layer ^3, 4, 5^. The literature suggests that this layer protects the alloy up to ~300°C, which is well above our highest *T*_x_ values of ~210°C. Above ~300°C the oxide layer is broken up by the elevated vapor pressure of Mg ^3, 4, 5^. The substrate is also not expected to have a significant effect. Since the amorphous phase is isotropic, the substrate cannot induce crystallographic texturing. Further, the characteristic length scale of the amorphous phase (~1 nm) is three orders of magnitude smaller than the total film thickness between 1,000 and 1,700 nm. Finally, the fragility is determined from the equilibrium liquid regime of the viscosity curve, where the relaxation time is shorter than the heating time scale. Thus, the liquid is expected to be in equilibrium and the measured fragility is unaffected by the heating rate. Here, a heating rate of 25 K/min was chosen, in order to closely match the heating rate of 20 K/min commonly used in DSC measurements.

**Viscosity Equation:** Equation (4) (main article) as derived by Eklund and Shkel ^6^ assumes Newtonian liquid behavior, which represents the behavior of metallic glasses and their liquids well, and a spherical deformation path, which is in agreement with the bubble shape after expansion to different heights, as verified for various samples under SEM. The equation further assumes a homogenous strain distribution across the bubble. In truth, however, a heterogeneous strain distribution is expected, with strain growing more quickly at the bubble’s apex than at its edges. The resulting discrepancy is expected to be negligible at small deformation heights below the hemispherical point (*h* = 1 mm). Even at the final expansion height, the majority of samples studied here were far from that hemispherical point (average final height ~300 to 400 μm). Thus, our extracted viscosity curves are affected only to a negligible degree. To the best of our knowledge, no analytical function equivalent to Equation (4) (main article) has been derived, that would properly account for a heterogeneous strain distribution. While Bloksma presents such an equation (see equation 4 in Supplementary of Reference ^7^), we have found the results to be less accurate, which we attribute to more debatable assumptions made about the deformation pathway.

**Uncertainty estimation:** Using the Gaussian law of error propagation, the uncertainty $U_{\log_{10} \left( \eta\right)}$ of $\log_{10} (\eta)$ is estimated at every point of the curve (see light green shaded area surrounding viscosity curve in Fig. 2.c.2 and 2.c.3):

|  | $U_{\log_{10} \left( \eta\right)}=\sqrt{\sum_{i} \left( \frac{\partial\log_{10} (\eta)}{\partial x_{i}}\cdot U_{i} \right)^{2}}$ | (1) |
| --- | --- | --- |

Here, $x_{i}$ are the respective variables with uncertainty $U_{i}.$ The partial derivatives are:

|  | $\frac{\partial\log_{10} (\eta)}{\partial p}=\frac{\log_{10} (e)}{\log_{10} (\eta)} \cdot\frac{1}{24 D_{0}}\cdot\frac{\left( r_{0}^{2}+h^{2} \right)^{3}}{r_{0}^{2}h^{2}}\cdot\frac{1}{\dot{h}}$ | (2) |
| --- | --- | --- |
|  |  |  |
|  | $\frac{\partial\log_{10} (\eta)}{\partial D_{0}}=\frac{\log_{10} (e)}{\log_{10} (\eta)} \cdot\frac{-p}{24 D_{0}^{2}}\cdot\frac{\left( r_{0}^{2}+h^{2} \right)^{3}}{r_{0}^{2}h^{2}}\cdot\frac{1}{\dot{h}}$ | (3) |
|  |  |  |
|  | $\frac{\partial\log_{10} (\eta)}{\partial r_{0}}=\frac{\log_{10} (e)}{\log_{10} (\eta)} \cdot\frac{p}{24 D_{0}}\cdot\frac{4r_{0}^{6}+6r_{0}^{4}h^{2}-2h^{6}}{r_{0}^{3}h^{2}}\cdot\frac{1}{\dot{h}}$ | (4) |
|  |  |  |
|  | $\frac{\partial\log_{10} (\eta)}{\partial h}=\frac{\log_{10} (e)}{\log_{10} (\eta)} \cdot\frac{p}{24 D_{0}}\cdot\frac{-2r_{0}^{6}+6r_{0}^{2}h^{4}+4h^{6}}{r_{0}^{3}h^{2}}\cdot\frac{1}{\dot{h}}$ | (5) |
|  |  |  |
|  | $\frac{\partial\log_{10} (\eta)}{\partial\dot{h}}=\frac{\log_{10} (e)}{\log_{10} (\eta)} \cdot\frac{-p}{24 D_{0}}\cdot\frac{\left( r_{0}^{2}+h^{2} \right)^{3}}{r_{0}^{2}h^{2}}\cdot\frac{1}{\left( \dot{h} \right)^{2}}$ | (6) |

The respective variable uncertainties are summarized in Supplementary Table 1:

**Supplementary Table 1: Variable uncertainty values.**

| $\boldsymbol{x}_{\mathbf{i}}$ | $\boldsymbol{U}_{\mathbf{i}}$ |
| --- | --- |
| $p$ | 1.5 mbar |
| $D_{0}$ | 5 nm |
| $r_{0}$ | 10 μm |
| $h$ | 10 μm |
| $\dot{h}$ | 0.05 μm/sec |

**Calorimetric literature data and interpolation to FIM compositions:** Values for *T*_g_ and *T*_x_ were compiled from the literature ^8, 9, 10, 11, 12^ for published compositions across the Mg-Cu-Y system, and are provided in Supplementary File “CalorimetricData_Literature.xlsx”. Values for the liquidus temperature *T*_L_ were compiled from the literature ^9, 13^ and the binary phase diagrams (ASM Phase Diagram Database), and are provided in Supplementary File “LiquidusData_Literature.xlsx”. Since these literature compositions do typically not coincide with the FIM compositions, *T*_g,Lit_ and *T*_x,Lit_ values (Figure 3b and d) and *T*_L_ values (Supplementary Figure 4) corresponding to each of the FIM compositions were determined by triangular interpolation from the respective three closest surrounding literature compositions. In combination, the error from this interpolation, along with some disagreement between the literature references, as well as our own FIM measurement error, introduce some scattering to the data shown in the comparison plots in Figure 3b and 3d. For *T*_g_, the literature values were corrected for better agreement based on the comparison between duplicate compositions, to achieve a closer agreement between the values reported by the different references.

**Complementary bulk sample fabrication for GFA:** To complement and verify the bulk GFA data collected from the literature, we prepared our own Mg-Cu-Y bulk samples using suction casting in wedge type geometry. Mg, Cu, and Y were jointly melted in graphite crucibles using inductive heating under flowing high-purity argon. The melt was then cast into a copper mold. The pyramid-shaped cavity had a 20° tip angle. Starting at a width of 8 mm, narrowing down to 0.5 mm, and continuing at this width in a straight channel of 10 mm length, through which suction was applied. During casting, the copper mold was cooled in a bath of liquid nitrogen. The resulting wedge samples were sectioned along the middle plane and characterized for amorphicity using SEM (Hitachi SU7000) and composition using EDX (Oxford AZtec). The critical casting thickness was determined as the sample width at the boundary between the amorphous and crystalline phase. From this set of amorphous bulk samples, we selected one at composition Mg_62.4_Cu_27.7_Y_9.9_ to record the DSC trace (TA DSC250, 25 K/min) displayed for illustration in Figure 2.c.2. Due to the compositional difference from Mg_64.1_Cu_28.1_Y_7.8_, the glass transition and crystallization slightly deviate between the DSC trace and the viscosity-temperature curve.

**Literature and bulk data for GFA:** Literature data were compiled from references ^8, 9, 10, 11, 12, 14^. For GFA, the data were corrected, cleaned, and curated as documented in Supplementary File “GFAData_Literature.xlsx”. These literature data were merged with our complementary bulk data, leading to the final set of GFA data shown in Figure 4b, as well as in Supplementary Figure 2 for the full Mg-Cu-Y range below, and as provided in Supplementary File “GFAData_Merged.xlsx”.

**
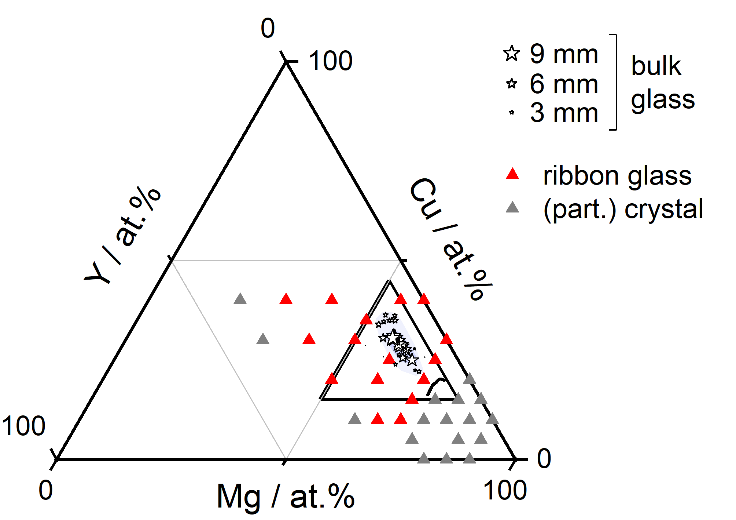
**

**Supplementary Figure 2:** Available curated GFA data shown for full Mg-Cu-Y composition range.

**Supplementary Figures**

**Correlation of *T*_g_ and *T*_x_ with pure constituent melting temperatures:** *T*_m,mean_ is the composition-weighted average melting temperature of the constituent elements. Here, *x*_i_ is the molar fraction and *T*_m,i_ is the melting temperature of element i:

|  | $T_{m,mean}=\sum_{i} x_{i}\cdot T_{m,i}$ | (7) |
| --- | --- | --- |

The values for *T*_m,i_ are ^15^ summarized in Supplementary Table 2:

**Supplementary Table 2:** Pure element melting temperatures in the Mg-Cu-Y system.

| element i | *T*_m,i_ / °C |
| --- | --- |
| Mg | 650 |
| Cu | 1085 |
| Y | 1522 |

Both *T*_g_ and *T*_x_ strongly correlate with *T*_m,mean_:


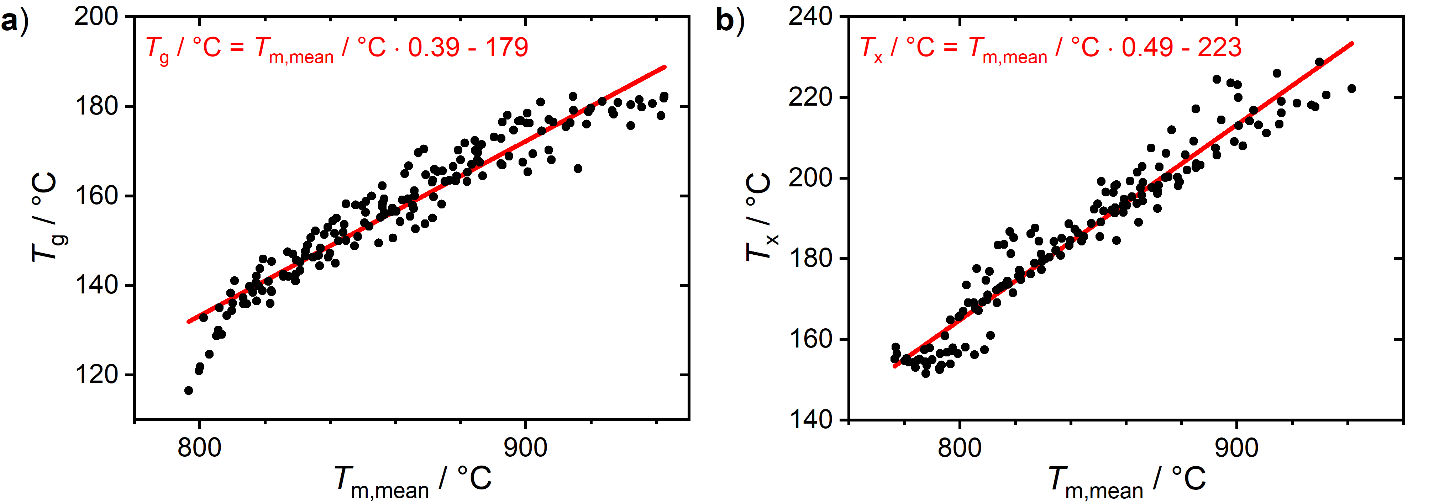


**Supplementary Figure 3:** Correlation of FIM measured **(a)** T_g_ and **(b)** T_x_ values with T_m,mean_.

**Liquidus temperature and melting point depression in Mg-Cu-Y:** From the compiled literature data, the liquidus temperatures corresponding to the compositions probed using FIM were interpolated, and the resulting composition map is shown:


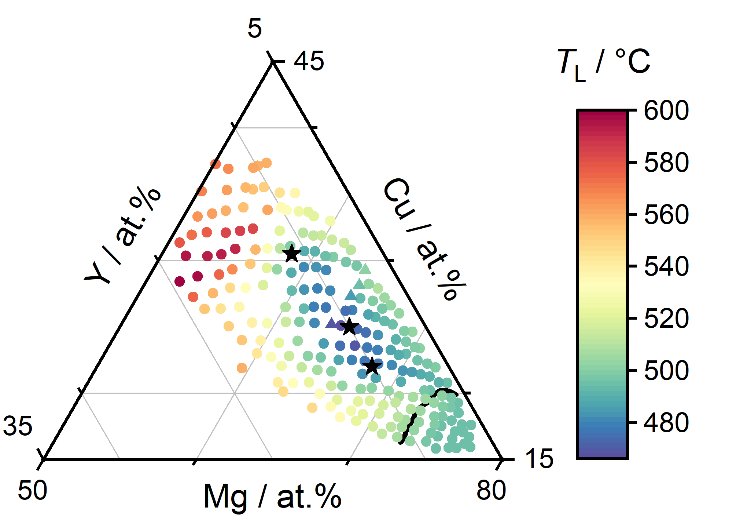


***Supplementary Figure 4:*** *Liquidus temperature distribution for FIM compositions. A deep eutectic is observed at Mg_65_Cu_25_Y_10_ (middle black star).*

The ratio *T*_L_ / *T*_m,mean_ then quantifies the degree of melting point depression:


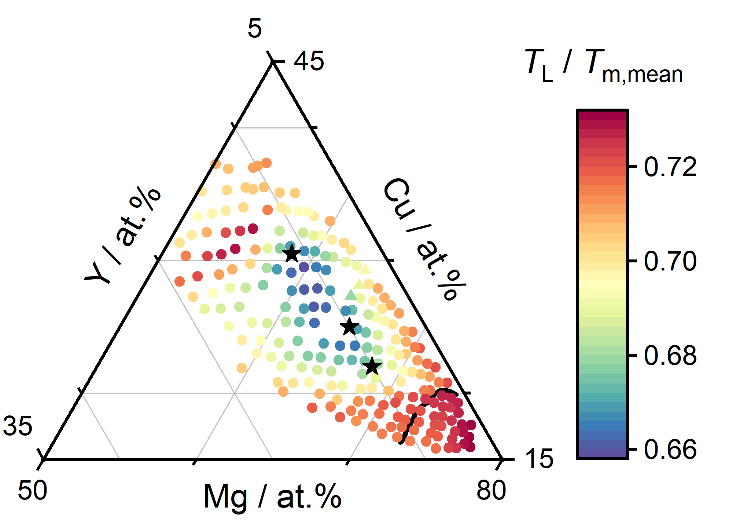


***Supplementary Figure 5:*** *Degree of melting point depression in Mg-Cu-Y. Based on the liquidus temperatures shown in Supplementary Figure 4.*

**Quantitative GFA prediction based on model by Greer et al.:** Greer et al. predict GFA according to ^16^:

|  | $T_{\mathrm{gu}}=T_{\mathrm{rg}}-m/505$ | (8) |
| --- | --- | --- |

In this perspective, *T*_rg_ is the starting point in determining GFA. *T*_rg_ is then adjusted by the contribution from the fragility parameter *m* to yield a corrected parameter *T*_gu_. The resulting composition map exhibits a GFA prediction that is qualitatively similar to the GFA prediction based on the model by Johnson et al.:


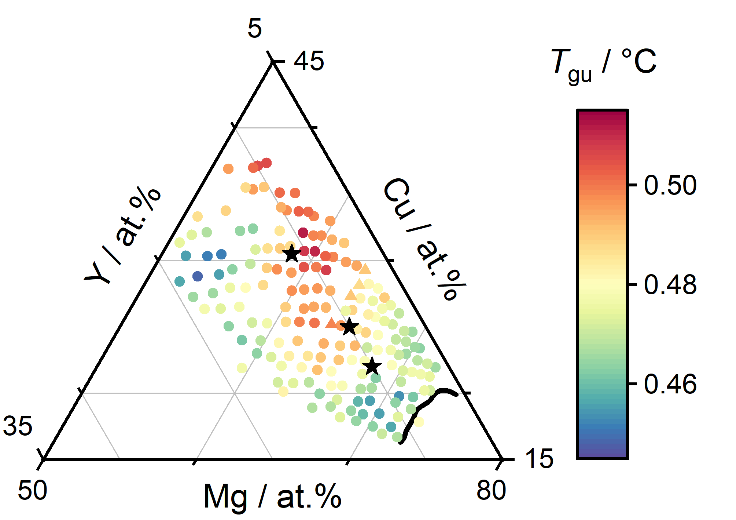


***Supplementary Figure 6:*** *GFA prediction using parameter T_gu_ based on model by Greer et al. ^16^.*

**Supplementary Discussion**

**Crystallization time in relaxation time units,** ${\hat{\boldsymbol{t}}}_{\mathbf{x}}$**:** $\hat{t}_{x}$ is an approximation to effectively eliminate the alloy-specific temperature dependence of crystallization times. The structural relaxation time quantifies the rate at which the entire ensemble of atoms undergoes rearrangement in structural relaxation and viscous flow. It therefore provides a readily available reference time scale for crystallization, which similarly involves comprehensive atomic rearrangement.

In detail, crystallization can be rather complicated on the atomic level. Indeed, close to *T*_g_ crystallization is growth-dominated and the nucleation rate dependence is negligible ^17^. However, in particular fragile liquids often show a decoupling of crystal growth rates from the viscosity upon approaching *T*_g_ ^16, 18, 19, 20^. Further, elemental diffusivities can differ by orders or magnitude depending on the atomic sizes, which is commonly known as the breakdown of the Stokes-Einstein Equation ^19, 21^. This is connected to the temperature dependence of alpha and beta relaxation, which are the mechanisms of atomic mobility enabling the larger scale processes of diffusion, viscous flow, and structural relaxation ^19, 20, 22^.

Nevertheless, $\hat{t}_{x}$ offers a useful indicator to independently compare crystallization complexity, since many metallic glasses do exhibit approximate proportionality over a small temperature interval close to *T*_g_ ^23, 24^. Amongst representative BMGs ^23, 24^, typical deviations from proportionality can be estimated at a factor of 1.5 or less, comparing between *T*_g_ and *T*_g_ + 30 K. Overall, $\hat{t}_{x}$ is ideally applied directly at *T*_g_, as the common reference state of all liquids, or slightly above *T*_g_, to ensure metastable equilibrium.

**Evaluating** ${\hat{\boldsymbol{t}}}_{\mathbf{x}}$ **from our FIM data:** Eq. (7) (main article) for $\hat{t}_{x}$ can be modified to quantify crystallization under heating conditions. As before, this is most valid for a small temperature range around *T*_g_, which makes $\hat{t}_{x}$ approximately temperature independent. For heating conditions across the interval between *T*_g_ and *T*_x_, which is typically small compared to *T*_g_ and *T*_L_, this allows us to define an equivalent expression for $\hat{t}_{x}$:

|  | $\hat{t}_{x}=\int_{t_{\tau=200 \sec}^{'}}^{t_{x}^{'}} \frac{dt}{\tau(t)}$ | (9) |
| --- | --- | --- |

Here, the time differential d*t* is normalized by the relaxation time $\tau(t)$ and integrated between the time points $t_{\tau=200 \sec}^{'}$ (close to glass transition) and $t_{x}^{'}$ (crystallization). We apply this equation to our FIM viscosity curves and calculate $\hat{t}_{x}$. For this, $\tau(T)\propto\eta(T)$ and $(\tau=200 s)\hat{=}(\eta={10}^{12}Pa\cdot s)$ are used to approximate the relaxation time from the measured viscosity, as illustrated in Figure 2c.3. The resulting $1/\tau\left( t \right)$curve is then integrated as function of the measurement time *t*. By performing this intergration on our own FIM curves, we obtain the composition map shown in Figure 4 in the main article.

Figure 4e illustrates crystallization complexity well, but some limitations are worth mentioning. On the one hand, our FIM data cross a temperature interval of up to 40 K beyond *T*_g_, possibly leading to a limited deviation from proportionality. On the other hand, due to the reduced stability of our film samples (~10 K reduced *T*_x_ values, Figure 3d), the $\hat{t}_{x}$ values vary by only two orders of magnitude. A variation by three or four orders of magnitude would be expected if the films displayed full stability. Finally, the relaxation time was approximated from our viscosity data assuming proportionality of $\tau(T)\propto\eta(T)$ and $(\tau=200 s)\hat{=}(\eta={10}^{12}Pa\cdot s)$. This constant of proportionality can vary between different alloy systems. However, isothermal viscosity equilibration measurements conducted on Mg_65_Cu_25_Y_10_ by Johnson et al. ^1, 25^ show that the relaxation time is indeed on the order of 200 seconds at *T*_g_, and the variation is expected to be small across our Mg-Cu-Y composition range.

**Compositional dependence of** ${\hat{\boldsymbol{t}}}_{\mathbf{x}}$**:** In Figure 4e, $\hat{t}_{x}$ overall varies by two orders of magnitude. In the vicinity of the bulk glass forming compositions, crystallization times are as high as 100 relaxation time units, indicating that crystallization is rather complex. Departing from this region, $\hat{t}_{x}$ decreases and correlates with lower GFA. This is particularly evident for those compositions which are already partially crystalline as-sputtered. For these, *R*_C_ must be ≥ 10^8^ K/s and crystallization almost follows a polymorphic pathway. Hence, they exhibit low GFA and their crystallization time is close to the relaxation time itself.

Based on the above, $\hat{t}_{x}$ suggests itself as a third parameter to predict GFA. For example, it provides a possible explanation why GFA may not reach a maximum of 25 mm at Mg_60_Cu_30_Y_10_, as predicted in Figure 4d: While the combination of high *T*_rg_ values with low fragility *m* values seems to suggest exceptional GFA here, $\hat{t}_{x}$ is reduced to only about 10 relaxation time units in this area. Therefore, alloys in the vicinity of Mg_60_Cu_30_Y_10_ crystallize quite readily, which limits their GFA. Altogether, these results suggest that a large $\hat{t}_{x}$ could be a valuable indicator of high crystallization complexity.

**Comparison between** ${\hat{\boldsymbol{t}}}_{\mathbf{x}}$ **and** $\boldsymbol{t}_{\mathbf{x}}\boldsymbol{(}\dot{\boldsymbol{T}}\boldsymbol{\approx25}\mathbf{K/min}\boldsymbol{)}$**:** $\hat{t}_{x}$ allows to eliminate the alloy-specific temperature dependence of crystallization kinetics. To highlight the improved perspective this offers, we compare $\hat{t}_{x}$ to $t_{x}(\dot{T}\approx25 K/min)$in Supplementary Figure 7a. The latter is the time to crystallization under heating conditions. It is determined from our FIM curves as $t_{\tau=200 \sec}^{'}- t_{x}^{'}$, i.e. as the difference between the bounds of integration used in Supplementary Equation (9).


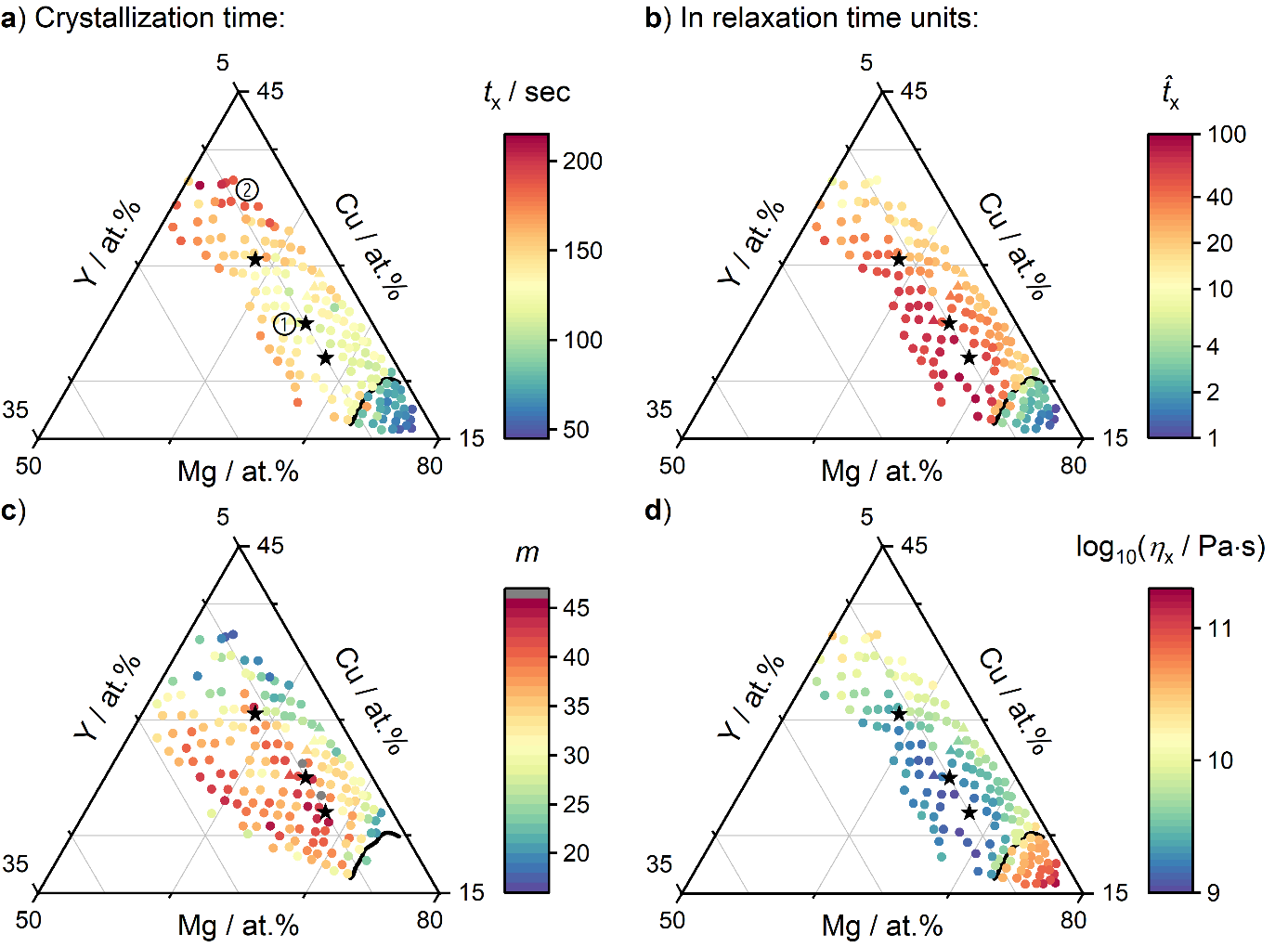


***Supplementary Figure 7: (a)*** *Crystallization time* $t_{x}$ *under heating conditions of ~25 K/min, compared to* ***(b)*** $\hat{t}_{x}$ *the crystallization time in relaxation time units. For reference,* ***(c)*** *shows the fragility m and* ***(d)*** *shows η_x_.*

For comparison we highlight two regions: The region around point ① exhibits high GFA, but the observed crystallization time of only ~100 seconds is comparatively low. Meanwhile, the region around point ② exhibits much lower GFA, but the crystallization time is about twice as long at ~200 seconds. These crystallization times seem to contradict the observed variation of GFA. This is misleading, however, because the crystallization time alone is meaningless if the underlying kinetic rates and their temperature dependence are not considered. Indeed, in region ① the fragility is significantly higher. Accordingly, *η*_x_, the viscosity at the point of crystallization, is an order of magnitude lower here, the associated kinetics are ten times faster, and the crystallization time is shorter. By contrast, the independent $\hat{t}_{x}$ indicator accounts for such temperature dependence, reveals that the crystallization complexity is actually higher in region ①, and strongly correlates with GFA.

**Illustration of the contributions to GFA:** Schematic providing a simplified overview of the individual effects of the three identified contributions to GFA. In particular, we envision that a larger $\hat{t}_{x}$ systematically shifts the whole TTT curve (red) to larger crystallization times. The crystallization complexity is not adequately captured by the fragility *m* (blue), which primarily reflects on the temperature-scaling of the overall kinetic rates, but not on the kinetic complexity of the processes involved in crystallization. Nor is it adequately captured by *T*_rg_ (green), which primarily quantifies the temperature interval across which crystallization must be avoided.


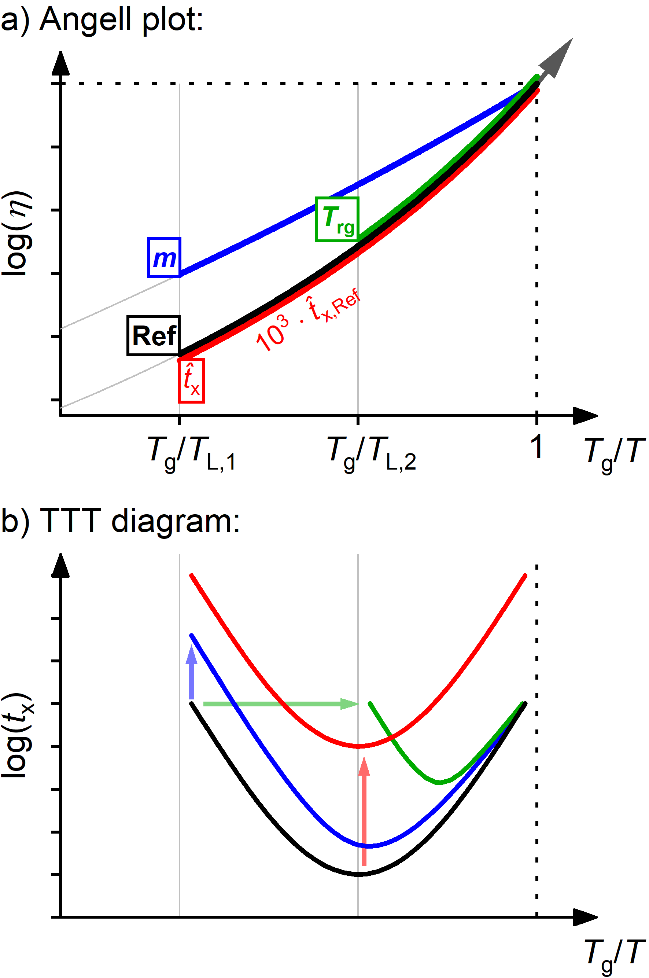


***Supplementary Figure 8:*** *Schematic illustration of the simplified effects of contributions m, T*_rg_*, and* $\hat{t}_{x}$*towards improved GFA. Compared to the reference glass (black curve), each colored curve represents an identical glass with enhanced GFA resulting from only one of the respective contributions.* ***a) Angell plot:*** *The colored curves correspond to identical cooling paths, differing only by a smaller fragility (blue), a larger T*_rg_ *(green), and a* $\hat{t}_{x}$ *which is orders of magnitude larger (red).* ***b) TTT diagram:*** *A smaller fragility increases the viscosity and thus the crystallization time for T*_g_*/T > 1 (blue). A larger T*_rg_ *scales the TTT curve into a smaller temperature interval with a higher minimum in crystallization time (green). A higher* $\hat{t}_{x}$ *shifts the entire TTT curve to longer crystallization times* $t_{x}$ *(red).*

**Supplementary References**

1. Busch R, Liu W, Johnson W. Thermodynamics and kinetics of the Mg 65 Cu 25 Y 10 bulk metallic glass forming liquid. *Journal of Applied Physics* **83**, 4134-4141 (1998).

2. Frey M, Busch R, Possart W, Gallino I. On the thermodynamics, kinetics, and sub-Tg relaxations of Mg-based bulk metallic glasses. *Acta Mater* **155**, 117-127 (2018).

3. Fournier V, Marcus P, Olefjord I. Oxidation of magnesium. *Surface and Interface Analysis: An International Journal devoted to the development and application of techniques for the analysis of surfaces, interfaces and thin films* **34**, 494-497 (2002).

4. Czerwinski F. The early stage oxidation and evaporation of Mg–9%Al–1%Zn alloy. *Corrosion Science* **46**, 377-386 (2004).

5. Baunack S, Subba Rao R, Wolff U. Characterization of oxide layers on amorphous Mg-based alloys by Auger electron spectroscopy with sputter depth profiling. *Analytical and bioanalytical chemistry* **375**, 896-901 (2003).

6. Eklund EJ, Shkel AM. Glass blowing on a wafer level. *Journal of Microelectromechanical Systems* **16**, 232-239 (2007).

7. Bloksma A. A calculation of the shape of the alveograms of some rheological model substances. *Cereal Chem* **34**, 126-136 (1957).

8. Kim SG, Inoue A, Masumoto T. High mechanical strengths of Mg–Ni–Y and Mg–Cu–Y amorphous alloys with significant supercooled liquid region. *Materials Transactions, JIM* **31**, 929-934 (1990).

9. Ma H, Shi L-L, Xu J, Li Y, Ma E. Improving glass-forming ability of Mg− Cu− Y via substitutional alloying: Effects of Ag versus Ni. *Journal of materials research* **21**, 2204-2214 (2006).

10. Inoue A, Nakamura T, Nishiyama N, Masumoto T. Mg–Cu–Y bulk amorphous alloys with high tensile strength produced by a high-pressure die casting method. *Materials Transactions, JIM* **33**, 937-945 (1992).

11. Inoue A, Kato A, Zhang T, Masumoto T. Mg–Cu–Y amorphous alloys with high mechanical strengths produced by a metallic mold casting method. *Materials transactions, JIM* **32**, 609-616 (1991).

12. Niikura A, Tsai A, Inoue A, Masumoto T. Chemical structural relaxation-induced embrittlement in amorphous Mg— Cu— Y alloys. *Journal of non-crystalline solids* **159**, 229-234 (1993).

13. Cheney J, Vecchio K. Prediction of glass-forming compositions using liquidus temperature calculations. *Materials Science and Engineering: A* **471**, 135-143 (2007).

14. Ding S*, et al.* Combinatorial development of bulk metallic glasses. *Nature Materials* **13**, 494 (2014).

15. Miracle D, Senkov O. A critical review of high entropy alloys and related concepts. *Acta Mater* **122**, 448-511 (2017).

16. Orava J, Greer Aá. Fast and slow crystal growth kinetics in glass-forming melts. *The Journal of chemical physics* **140**, 214504 (2014).

17. Schroers J, Wu Y, Busch R, Johnson W. Transition from nucleation controlled to growth controlled crystallization in Pd43Ni10Cu27P20 melts. *Acta Mater* **49**, 2773-2781 (2001).

18. Nascimento MLF, Dutra Zanotto E. Does viscosity describe the kinetic barrier for crystal growth from the liquidus to the glass transition? *The Journal of chemical physics* **133**, 174701 (2010).

19. Yu HB, Samwer K, Wu Y, Wang WH. Correlation between $\ensuremath{\beta}$ Relaxation and Self-Diffusion of the Smallest Constituting Atoms in Metallic Glasses. *Physical Review Letters* **109**, 095508 (2012).

20. Debenedetti PG, Stillinger FH. Supercooled liquids and the glass transition. *Nature* **410**, 259-267 (2001).

21. Masuhr A, Waniuk TA, Busch R, Johnson WL. Time Scales for Viscous Flow, Atomic Transport, and Crystallization in the Liquid and Supercooled Liquid States of ${\mathrm{Zr}}_{41.2}{\mathrm{Ti}}_{13.8}{\mathrm{Cu}}_{12.5}{\mathrm{Ni}}_{10.0}{\mathrm{Be}}_{22.5}$. *Physical Review Letters* **82**, 2290-2293 (1999).

22. Volkert C, Spaepen F. Crossover relaxation of the viscosity of Pd40Ni40P19Si1 near the glass transition. *Acta Metallurgica* **37**, 1355-1362 (1989).

23. Gun B, Laws KJ, Ferry M. Static and dynamic crystallization in Mg–Cu–Y bulk metallic glass. *Journal of Non-Crystalline Solids* **352**, 3887-3895 (2006).

24. Bryn Pitt E, Kumar G, Schroers J. Temperature dependence of the thermoplastic formability in bulk metallic glasses. *Journal of Applied Physics* **110**, 043518 (2011).

25. Zheng Q, Xu J, Ma E. High glass-forming ability correlated with fragility of Mg–Cu (Ag)–Gd alloys. *Journal of Applied Physics* **102**, 113519 (2007).
